# Neutrophils: Innate Effectors of TB Resistance?

**DOI:** 10.3389/fimmu.2018.02637

**Published:** 2018-11-14

**Authors:** Elouise E. Kroon, Anna K. Coussens, Craig Kinnear, Marianna Orlova, Marlo Möller, Allison Seeger, Robert J. Wilkinson, Eileen G. Hoal, Erwin Schurr

**Affiliations:** ^1^DST-NRF Centre of Excellence for Biomedical Tuberculosis Research, South African Medical Research Council Centre for Tuberculosis Research, Division of Molecular Biology and Human Genetics, Faculty of Medicine and Health Sciences, Stellenbosch University, Cape Town, South Africa; ^2^Wellcome Centre for Infectious Diseases Research in Africa, Institute of Infectious Disease and Molecular Medicine, University of Cape Town, Cape Town, South Africa; ^3^Infection and Immunity Division, Walter and Eliza Hall Institute of Medical Research, Parkville, VIC, Australia; ^4^Division of Medical Biology, Faculty of Medicine, Dentistry and Health Sciences, University of Melbourne, Melbourne, VIC, Australia; ^5^Program in Infectious Diseases and Immunity in Global Health, The Research Institute of the McGill University Health Centre, Montreal, QC, Canada; ^6^McGill International TB Centre, McGill University, Montreal, QC, Canada; ^7^Departments of Medicine and Human Genetics, McGill University, Montreal, QC, Canada; ^8^Department of Medicine, Imperial College London, London, United Kingdom; ^9^The Francis Crick Institute, London, United Kingdom

**Keywords:** *Mycobacterium*, tuberculosis, inflammation, NETs, antimicrobial, protection, necrosis

## Abstract

Certain individuals are able to resist *Mycobacterium tuberculosis* infection despite persistent and intense exposure. These persons do not exhibit adaptive immune priming as measured by tuberculin skin test (TST) and interferon-γ (IFN-γ) release assay (IGRA) responses, nor do they develop active tuberculosis (TB). Genetic investigation of individuals who are able to resist *M. tuberculosis* infection shows there are likely a combination of genetic variants that contribute to the phenotype. The contribution of the innate immune system and the exact cells involved in this phenotype remain incompletely elucidated. Neutrophils are prominent candidates for possible involvement as primers for microbial clearance. Significant variability is observed in neutrophil gene expression and DNA methylation. Furthermore, inter-individual variability is seen between the mycobactericidal capacities of donor neutrophils. Clearance of *M. tuberculosis* infection is favored by the mycobactericidal activity of neutrophils, apoptosis, effective clearance of cells by macrophages, and resolution of inflammation. In this review we will discuss the different mechanisms neutrophils utilize to clear *M. tuberculosis* infection. We discuss the duality between neutrophils' ability to clear infection and how increasing numbers of neutrophils contribute to active TB severity and mortality. Further investigation into the potential role of neutrophils in innate immune-mediated *M. tuberculosis* infection resistance is warranted since it may reveal clinically important activities for prevention as well as vaccine and treatment development.

## Introduction

Not all individuals exposed to *Mycobacterium tuberculosis* become infected as inferred by a lack of T cell memory response to *M. tuberculosis* antigens. Moreover, these individuals do not develop signs and symptoms suggestive of ‘active tuberculosis' (TB). The majority of *M. tuberculosis* infected individuals remain asymptomatic with what is known as latent tuberculosis infection (LTBI).

Only 5–15% of those infected will progress to active TB disease, given they have no underlying co-morbidity which would increase their risk further (1, 2). This resulted in an estimated 10.4 million new cases and 1,674 million TB deaths reported in 2016 (3). The remaining 85–95% of persons with LTBI who do not develop disease indicates that the majority of those infected have a natural immunity to prevent the progression from infection to disease. Similarly, certain individuals who are highly exposed, never develop evidence of infection. This suggests that they are naturally resistant to *M. tuberculosis* and can prevent infection via an innate immune response prior to adaptive immune cell priming, and are known as “innate resisters” (4). The mechanisms that underlie the resistance to infection in persons of the “innate resister” phenotype are not fully known. In the present article, we explore the possible contribution of neutrophils to innate infection resistance.

### Evidence of *M. tuberculosis* infection

LTBI is defined as the presence of *M. tuberculosis*-specific T-cell sensitization in the absence of clinical signs and symptoms of TB. Host sensitization is used as a proxy for this assumed latent *M. tuberculosis* infection in human hosts and is measured by reactivity to mycobacterial antigens using the tuberculin skin test (TST) or interferon-γ (IFN-γ) release assays (IGRAs). The TST is performed by injecting purified protein derivate (PPD) intradermally (5). A delayed-type hypersensitivity reaction occurs if the host is reactive to *M. tuberculosis* antigens. Due to the limited *M. tuberculosis* specificity of TST, more specific *in vitro* blood-based assays (T-SPOT.*TB* and QuantiFERON-TB Gold) were developed using early secretory antigen target-6 (ESAT-6), culture filtrate protein 10 (CFP-10), and TB-7.7 as *M. tuberculosis* antigens. These assays measure the *ex-vivo* IFN-γ release by T cells in response to the aforementioned *M. tuberculosis* peptide antigens (6). A disadvantage of TST and IGRA for the diagnosis of infection is that they are unable to distinguish between an amnestic response and persistent infection. It is therefore possible that an unknown proportion of persons who test positive in the immune assays are no longer infected with *M. tuberculosis*. Conversely, persons who test negative in the immune assays may be (i) not sufficiently exposed to *M. tuberculosis*, (ii) anergic to *M. tuberculosis* antigens used in the assays, or (iii) exposed but able to clear *M. tuberculosis* infection without triggering the onset of acquired anti-*M. tuberculosis* immunity.

### Natural immunity against *M. tuberculosis*

While the lack of a direct assay for the determination of current infection complicates studying resistance to infection, multiple lines of evidence support human variability in resistance to infection with *M. tuberculosis*. Historical epidemiological studies have long supported the concept of infection resistance as a *bona-fid*e biological phenotype. During an outbreak on a US naval ship, 66 sailors shared a cabin with 7 sailors who had active TB. Of the 66 sailors, 13 (20%) remained TST negative after 6 months (7). Fifty-seven (55%) of 104 elderly residents with a previously TST negative result remained uninfected after being exposed for at least 12 months to a fellow resident with sputum positive TB (8). An average of 50% of close contacts of TB patients develop positive TST or IGRA tests in overcrowded living conditions or household contact studies (9, 10). In Uganda only 4.1% of adults (age > 15 years old) with close household contacts remained PPD negative (<10 mm for HIV- adults, <5 mm for all HIV+) over a 2 year follow up period (11). Other studies done in individuals in environments with high exposure to *M. tuberculosis*, show that 10–20% do not become TST/IGRA positive (12–14). In South African goldminers who have a documented high exposure to *M. tuberculosis* and an estimated LTBI prevalence of 89% in 2006, 13% of the HIV-negative participants had a TST = 0 mm response (15). Together, these studies suggest that 5–20% of the population may possess resistance to *M. tuberculosis* infection.

Molecular genetics studies support the concept of resistance to *M. tuberculosis* infection. In a highly TB endemic area in South Africa 20% of the highly exposed population remained TST negative which was stringently defined as TST = 0 mm. This phenotype is linked to a major locus, *TST1*, which represents T cell-independent *M. tuberculosis* infection resistance (13). A genome-wide association study in HIV-infected persons identified a locus on chromosome region 5q31.1 in proximity of *IL9* which significantly associates with TST positivity (16). In addition, the study replicated associations in the region of *TST1* as well as on chromosome regions 2q21-2q24 and 5p13-5q22 that had been identified by genome-wide linkage analysis of Ugandan families (13, 16, 17). Current genetic evidence suggests that the resistance phenotype is likely due to a combination of genetic variants synergistically contributing to the phenotype rather than a single genetic variant.

## The heterogeneous nature of neutrophils

It is tempting to speculate that neutrophils of individuals who exhibit *M. tuberculosis* infection resistance are a unique subset of cells genetically or epigenetically programmed to control infection and inflammation. Epigenetic reprogramming of neutrophils offers an attractive avenue of investigation as neutrophils show increased variability in both gene expression and DNA methylation compared to phenotypically naïve T-cells and classic monocytes (18). This observation supports the concept of physiologically distinct inter-individual neutrophil populations.

Different intra-individual neutrophil subsets have also been defined in multiple studies investigating various diseases including cancer, systemic lupus erythematosus (SLE), TB, and HIV-1 (19–23). However, the heterogeneous nature of neutrophils with subsets displaying functional as well as phenotypic differences is still under debate and most subsets remain incompletely defined and phenotyped (20, 24–29).

Genetic variants, which underlie epigenetic and transcriptional variability, also contribute to differences in neutrophil activity. For example, 21 neutrophil genes showed significant differences in expression levels between males and females while a SNP in *SELL*, which encodes the CD62L receptor, strongly influenced expression levels of CD62L cell receptors on neutrophils (18, 30). Not surprisingly, genes of the inflammasome pathway are significantly enriched in neutrophils and play an important role in the regulation of interleukin 1 (IL-1)-dependent cytokine production (31). In murine studies, IL-1 deficiency predisposes to a lack of *M. tuberculosis* infection control and non-resolving inflammation (32). During persistent infections, such as active TB, inflammasome activation correlates with pathology (33, 34). Taken together, these data suggest that if neutrophils contribute to *M. tuberculosis* infection resistance the effector mechanisms involved are likely to be under both genetic and epigenetic regulation. However, at least some of the underlying variability may be ascribed to the inherent difficulties in working with these cells since they cannot be cryopreserved, are easily activated and are short-lived (35, 36). Possible genetic variability is further highlighted by the conflicting results published around the role of neutrophils in *M. tuberculosis* infection.

## Neutrophils in *M. tuberculosis* infection and disease

**Figure 1:**
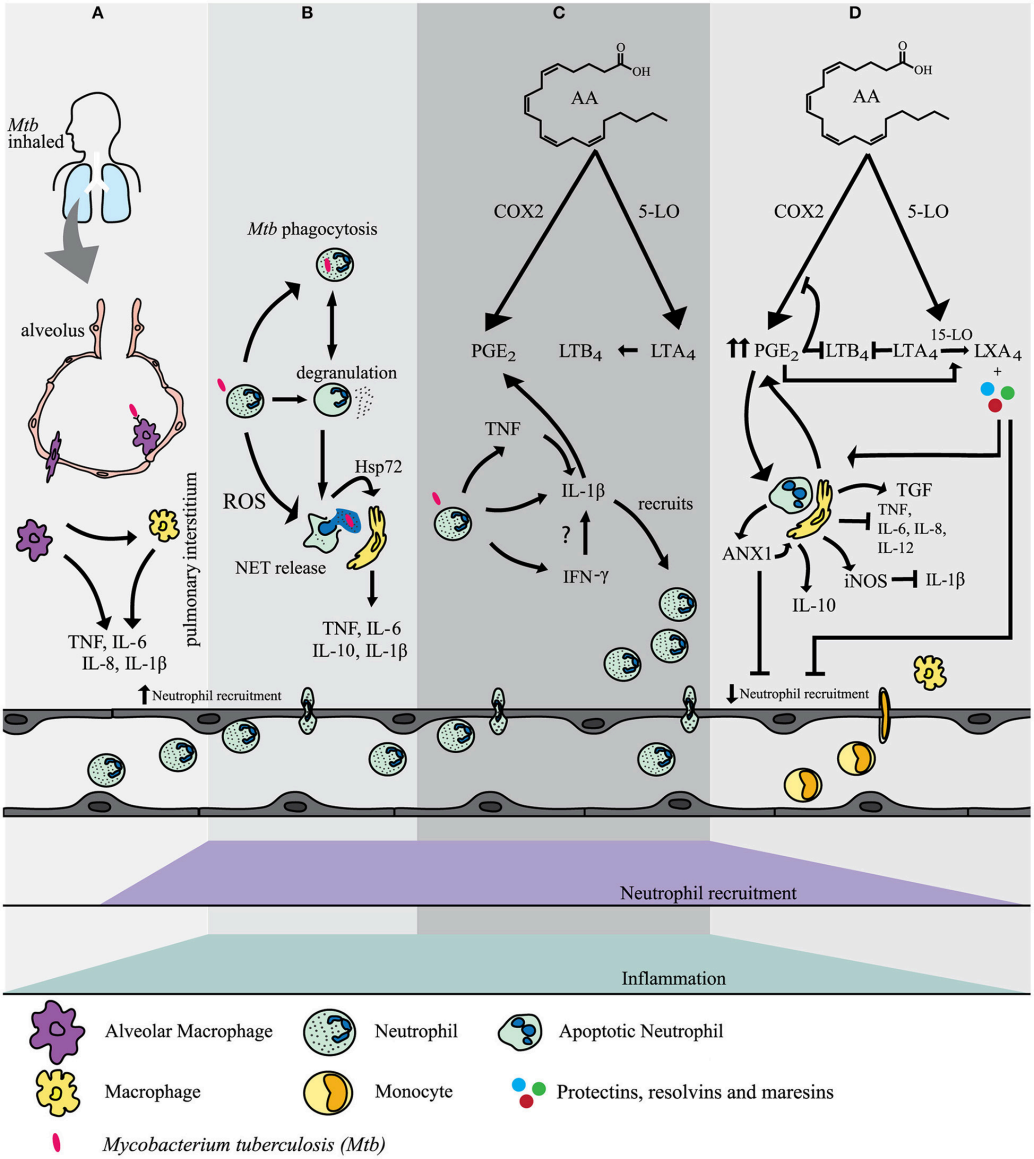
**(A)** Alveolar macrophages (AM) are the first cells to encounter *M. tuberculosis* after inhalation of the bacillus. Acute pro-inflammatory signals are released by AM and local tissue macrophages to recruit neutrophils to the site of infection.**(B)** Neutrophils use a variety of mechanisms to mediate *M. tuberculosis* infection. These included phagocytosis, degranulation, ROS formation and NET release. NETs transfer Hsp72 to adjacent macrophages inducing a pro-inflammatory response.**(C)** Interaction of recruited neutrophils with *M. tuberculosis* mediates the activation of several pathways which contribute to inflammation and clearance of *M. tuberculosis* infection. Interleukin-1β (IL-1β) release is mostly mediated in an inflammasome dependent manner. Tumour necrosis factor (TNF) induces NF-κB which mediates the induction of gene expression of IL-1β in neutrophils. Interferon-γ (IFN-γ) may also regulate the release of IL-1β. IL-1β is a key player in mediating the release of prostaglandin E_2_ (PGE_2_) and leukotriene B_4_ (LTB_4_) both of which contribute to inflammation and the recruitment of neutrophils. **(D)** PGE_2_ eventually becomes a stop signal and has a negative feedback on cyclo-oxygenase-2 (COX-2) and 5-lipoxygenase (5-LO). The production of lipoxin A4 (LXA4) is favoured. In addition, AnnexinA1 (ANX1) stimulates IL-10 release by macrophages. Neutrophils express inducible nitric oxide synthase (iNOS) which has a further negative feedback on IL-1β release. The net effect is an increase in neutrophil apoptosis and clearance by tissue macrophages. More macrophages are recruited and further neutrophil recruitment is inhibited and inflammation is resolved.

*M. tuberculosis* is an airborne pathogen and is transmitted via the aerosol inhalation of transmitted droplets containing the bacteria from an infected individual. *M. tuberculosis* enters the airways and reaches the pulmonary alveolus where some of the first cells encountered are resident alveolar macrophages (AM) (37) which release pro-inflammatory cytokines tumor necrosis factor (TNF), IL-6, IL-1α, and IL-1β (38). If this first line of defense fails, *M. tuberculosis* enters the pulmonary interstitial tissue by either using the infected AM as a host vehicle to migrate or by infecting the epithelium or pneumocytes (2). Acute inflammatory signals are released and the other phagocytes are recruited to the site of infection. Local tissue macrophages recognize *M. tuberculosis* by Toll-like receptors (TLR) and are also activated to release pro-inflammatory cytokines including TNF, IL-6, and IL-1β (39, 40) (Figure 1A).

Neutrophils are some of the first phagocytes recruited from the pulmonary vasculature to the pulmonary interstitium (41). Multiple receptors (including TLRs and C-type lectins receptors (CLRs) and cytokine receptors) have been implicated in the interaction between neutrophils, *M. tuberculosis* and pro-inflammatory cytokines (42–46). Upon exposure to *M. tuberculosis* neutrophil blood counts in human pulmonary TB (PTB) contacts are initially higher than in unexposed control subjects and subside after 6 weeks (47). Interestingly, low neutrophil counts are associated with IGRA positivity in TB contacts (47). The initial neutrophil peak seen in TB contacts, implicates neutrophils in the acute inflammatory response to *M. tuberculosis*.

Individuals in contact with patients with pulmonary TB are less likely to be infected with *M. tuberculosis* if they have higher peripheral blood neutrophil counts (47). One hour after *in vitro* infection with virulent *M. tuberculosis* and stimulation with TNF, neutrophils suppressed the growth of the inoculum by 50–95% (48). Unstimulated neutrophils inhibit on average 40.6% of the growth of the *M. tuberculosis* inoculum. Interestingly, there was significant variability in this mycobactericidal capacity between donor neutrophils. Neutrophils from some donors were capable of inhibiting *M. tuberculosis* growth spontaneously while, despite the addition of TNF or IFN-γ, others were not. Neutrophil-depleted whole blood had a 3.1 fold decreased capacity to control *M. tuberculosis* infection *ex vivo* (47). This finding was recently confirmed and highlights the importance of neutrophils in *M. tuberculosis* infection (49). Granulocyte (CD15+) depleted blood does not control *M. tuberculosis* infection as efficiently as blood depleted of CD4+, CD8+, or CD14+ cells. Addition of viable CD15+ granulocytes significantly improved *M. tuberculosis* control (49).

However, infection in highly susceptible strains of mice shows the detrimental effect of uncontrolled neutrophil recruitment on TB infection and inflammation control and eventually an increase in TB disease severity (50). Most studies concur that neutrophils are final mediators of lung damage and disease (51–53). C57BL/6 mice with neutrophil and monocyte derived-cells lacking *Atg5* succumb after 30–40 days post *M. tuberculosis* infection due to a massive influx of neutrophils, and increased lesion number and bacterial load, that is not observed in wild type mice (54, 55). Whilst ATG5 is normally associated with autophagy, the neutrophilic influx associated with premature death was independent of any autophagic response. Granulomas of various susceptible mouse strains contain a substantial number of necrotic neutrophils (53, 56–58) in comparison to more “resistant” mouse strains showing only scattered neutrophils and little or no necrosis (59).

In humans, as in the mouse model, necrotic neutrophils are unable to control *M. tuberculosis* infection (49). Phagocytosis of *M. tuberculosis*-induced necrotic neutrophils by macrophages promotes bacterial growth (60, 61). *M. tuberculosis* mostly remains encapsulated in apoptotic neutrophils (60). This enables fusion of neutrophil granular contents with macrophage lysosomes after efferocytosis of the apoptotic neutrophil by the macrophage (60). The neutrophil membranes surrounding *M. tuberculosis* prevent direct contact between the bacillus and the macrophage phagosomal membrane thus preventing *M. tuberculosis* inhibition of phagolysosome maturation (60). However, during neutrophil necrosis, *M. tuberculosis* is released from the disintegrated phagosome and enters the phagocytosing macrophage as extracellular bacteria (60). Once phagocytosed by a macrophage, the bacillus is able to evade phagolysosomal fusion in the macrophage and mycobacterial growth is promoted (60, 61).

Necrotic neutrophils added to whole blood increased the metabolism of *M. tuberculosis*, as measured by mycobacterial luminescence, and released IL-10 as well as growth factors, granulocyte- and granulocyte macrophage-colony-stimulating factors (G-CSF and GM-CSF), and the monocyte chemotactic protein chemokine ligand 2 (CCL2) (49). The predominant role of these molecules is to attract and prime more cells (49). G-CSF supports the growth and proliferation of neutrophils and their precursors (62). GM-CSF has the potential to act on earlier progenitor cells than G-CSF and therefore neutrophil progenitors as well as monocytes proliferate (63). G-CSF and GM-CSF not only drive the increased production of neutrophils and monocytes but also have the ability to indirectly affect neutrophil function and phenotype (64–68). Both of these growth factors delay neutrophil apoptosis and “prime” neutrophils for enhanced oxidative effects that can lead to tissue destruction (67, 69). In a setting where *M. tuberculosis* induces necrotic cell death, the newly released cells would undergo the same cycle of necrosis, release tissue damaging substances, recruit more cells, and contribute to continuous inflammation as seen in TB disease, with neutrophilia being an independent predictor of TB mortality (70).

### Neutrophil mechanisms to clear *M. tuberculosis* infection

Despite the involvement of neutrophils in tissue damage in late stage clinical TB, they display an inter-individual ability to control *M. tuberculosis* infection. Neutrophils can use oxidative and non-oxidative mechanisms to clear *M. tuberculosis* infection. Both mechanisms are involved in either the direct clearance of *M. tuberculosis* or in the mediation thereof.

#### Oxidative mechanisms

Neutrophils are primed or activated by *M. tuberculosis* and pro-inflammatory cytokines, which in turn triggers degranulation and respiratory burst (71–79). Proteases (e.g., elastase, cathepsin G, and protease 3), hydrolyses, antimicrobial peptides and oxidants are released. The oxidants mediate tissue breakdown by activating matrix metalloproteinases (MMPs) (80, 81). These effectors do not discriminate between pathogen and host tissue and collateral damage is inevitable.

Neutrophil-produced reactive oxygen species (ROS) have been shown to drive *M. tuberculosis*-induced necrosis (60). Inhibiting myeloperoxidase (MPO) derived ROS prevents neutrophil necrosis and improves efferocytosis of these cells by macrophages and therein the control of *M. tuberculosis* growth (60). Similarly, chronic granulomatous disease (CGD) neutrophils are protected from necrosis after infection with *M. tuberculosis* (79)*. One* would therefore expect an improved control of *M. tuberculosis* infection in CGD patients who are characterized by an inability to produce ROS but this does not always seem to be the case (82). Indeed, CGD patients are more susceptible to active TB supporting the possible role of neutrophils in mediating *M. tuberculosis* infection resistance (83–86). This view is supported by multiple studies that have shown neutrophils to be protective in control of early infection (47–49).

The NOX2 complex is an isoform of the large family of NAPDH oxidases (NOX) and is found in phagocytes including neutrophils (87, 88). It is an enzyme that is involved in infection and inflammation control and is activated by neutrophil chemotactic factors such as IL-8 and leukotriene B4 (LTB4) (88, 89). Hydrogen peroxide (H_2_O_2_) that is produced during respiratory burst contributes to neutrophil migration and subsequently retention at the site of infection (89). CGD patients have impaired neutrophil accumulation, in contrast to the increase in granuloma formation seen in CGD (89). Inflammatory leukotrienes are released by neutrophils in CGD patients but due to a lack of ROS there is a lack of degradation of these leukotrienes and delayed clearance of inflammation (44, 89, 90).

Reactive oxygen species have been shown to affect transcription factors such as NF-κβ (91, 92) which mediates the induction of IL-1β and IL-8 expression. However, CGD shows that NF-κβ activation is independent of ROS and is also mediated by TNF and IL-1 (93, 94) and so neutrophils in these individuals are still able to release these pro-inflammatory factors and uncontrolled chronic inflammation ensues (95, 96). Pro-inflammatory mediators alone, such as leukotrienes and IL-1β, are not enough to control infection and it is likely that the overproduction thereof augments the lack of *M. tuberculosis* infection control in CGD patients (44).

*M. tuberculosis* is relatively resistant to the bactericidal effects of H_2_O_2_ mediated by DNA damage (97)_._ However, even if ROS does not have a direct bactericidal effect on *M. tuberculosis*, it still amplifies the neutrophil antimicrobial response. It does this by activating the formation of neutrophil extracellular traps (NET, discussed in 3.1.3), stimulating the release of pro-inflammatory cytokines such as TNF and macrophage inflammatory protein 2 (MIP-2), as well as decondensed DNA to which the contents of cytoplasmic granules adhere in a net-like structure (98–100). This is extensively reviewed by Deffert et al. (44).

#### Non oxidative mechanisms

Neutrophil granules can fuse with the phagolysosome, degranulate and release antimicrobial peptides (AMPs) (Figure 1B). Antimicrobial peptides (AMPs) are classified according to their amino acid motif and structure. Three classes are found in humans: defensins, cathelicidins, and histatins (101–103). Neutrophils contain α-defensins in azurophilic granules and cathelicidin LL-37 in specific granules, as well as other neutrophil specific AMPs as will be discussed below (101, 104). Macrophages can traffic phagocytosed apoptotic neutrophil debris, including neutrophil granules, to endosomes. The purified neutrophil granules in the endosomes fuse with the macrophage phagosome in which the *M. tuberculosis* bacillus resides. This mechanism of cell-cell cooperation provides an effective antimicrobial response to *M. tuberculosis* (105). Although this efferocytosis occurs between macrophages and apoptotic neutrophil debris, it is not known whether alveolar macrophages do the same. AMPs can also be associated extracellularly with NETs and facilitate in the clearing of microbial infection.

a. AMPs in azurophilic granules

Azurophilic granules are poorly mobilized in response to *M. tuberculosis* infection. Pathogenic mycobacteria block the fusion of azurophil granules with the phagosome and consequentially unlike specific granules they are unable to release their contents in to the phagosome for antimicrobial effect (106). However, azurophilic proteins obtained from apoptotic neutrophil debris, increase macrophage ability to restrict *M. tuberculosis* growth either by direct action or by lysosome fusion with the maturation-arrested mycobacterial phagosome in the macrophage (107).

-Defensins: Human neutrophil peptide 1 (HNP-1), one of four α-defensins found in the primary or azurophilic granules of neutrophils (101) has the ability *in vitro* to reduce the growth of *M. tuberculosis* in culture as well as within macrophages (105, 108, 109). Furthermore, HNP-1 also shows *in vivo* antimycobacterial activity in mice (110).

-Azurocidin: Defensin depleted azurophilic granules at 100 μg/ml were shown to restrict the growth of 55% of *M. tuberculosis* in culture after 24 h of incubation. However, the specific role of azurocidin in *M. tuberculosis* infection remains unclear (107).

-Cathepsins: *M. tuberculosis* infection decreases cathepsin gene expression in macrophages, with a parallel decrease in cathepsin protein levels (111). Genetic linkage and association studies have previously implicated cathepsin Z in susceptibility to TB (112, 113). A likely alternative source of cathepsin for macrophages is through the phagocytosis of apoptotic neutrophil material. Uptake of liposomal encapsulated cathepsin G and neutrophil elastase (NE) by alveolar macrophages in mice improves antimicrobial activity against *Mycobacterium bovis* bacillus Calmette-Guérin (BCG) (114).

b. Specific granules

-Cathelicidin: Neutrophils produce LL-37, the 37 amino acid biologically active C-terminal domain cleaved from the human cathelicidin propeptide (hCAP18) by proteinase 3, when infected with *M. tuberculosis* (115). LL-37 has been shown to restrict growth of *M. tuberculosis* in neutrophils (47). Similarly it restricts growth of *M. tuberculosis* in infected macrophages when hCAP18 is exogenously activated by neutrophil proteinase 3, which has only a low level of constitutive expression in macrophages (107, 116).

c. Gelatinase granules

-Lipocalin 2: Lipocalin 2 binds mycobacterial siderophores which scavenge iron for the bacillus in iron-limiting conditions (47). Lipocalin 2 has a greater mycobacterial suppressive effect (60%) in an iron-depleted broth (10 nM iron) compared to iron-replete broth (150 μM Fe) of 45%. It may be more effective in the phagolysosome where the molar ratio to siderophores would be higher.

d. Neutrophil cytoplasmic proteins

Calprotectin (S100A8/S110A9): Calprotectin is known as a damage-associated molecular pattern (DAMP) molecule and is a heterodimer of S100A8/A9. It sequesters free zinc and limits mycobacterial growth (107, 117). *M. tuberculosis* infection induces S100A8/A9 proteins. This is associated with neutrophil accumulation and exacerbated inflammation (52, 118).

#### NET formation

During NETosis, neutrophils release their DNA contents coated in cytoplasmic and granular proteins to trap and possibly clear invading pathogens (119, 120). NETosis is an alternative form of cell death, different to apoptosis and necrosis, and mediated by phagocytosis and the generation of ROS by NADPH oxidase in *M. tuberculosis* infection (121, 122). Once activated, neutrophils lose their lobulated morphology (123). The nuclear membrane initially remains intact whilst the chromatin (histones and DNA) starts to decondense. Once the nuclear and granular membranes rupture, the decondensed chromatin comes into contact with the granular as well as cytoplasmic components of the cell. The NET components are released extracellularly when the cell membrane breaks (122). The most abundant non-histone protein in NETs is NE (124). In addition to this, NETs contain myeoloperoxidase (MPO) as well as other proteins from intracellular neutrophil organelles. These include substances from the primary neutrophil granule (cathepsin G, defensins, BPI-bactericidal substance), the secondary neutrophil granule (alcaic phosphatase, lactoferrins, lysozyme, cathelicidins, collagenase), tertiary granules [gelatinase, matrix metalloproteinase 9 (MMP-9)]; and catalase from peroxisomes (125–128). Other components include calprotectin, constituents of the neutrophil cytoskeleton and glycolytic enzymes (125, 128).

Although *M. tuberculosis* has been shown to induce NETosis, no experimental evidence exists that NET formation improves resolution of *M. tuberculosis* infection (129). However, the AMP NET components have been shown to restrict *M. tuberculosis* growth as discussed earlier. Also neutrophils can assist macrophages to clear *M. tuberculosis* infection. During infection, NET formation and *M. tuberculosis*-induced apoptosis occur independently. *M. tuberculosis*-induced NETs transfer the danger signal heat shock protein 72 (Hsp72) to adjacent macrophages (121). This interaction induces a pro-inflammatory response in macrophages leading to the release of IL-6, TNF, IL-1β, IL-10. In addition to these cytokines, calprotectin is released from the neutrophil cytoplasm into NETs (130). IL-10 is also released as part of the anti-inflammatory regulatory response via inhibiting IFN-γ and TNF production and downstream Th1 responses (121). It is possible that NETs play a role in trapping and localizing the infection. The sequestration of AMPs in the NET structures may also increase their effective concentrations. Furthermore; NETs contain the release of cellular contents to prevent distal tissue destruction (121, 123). Hence, NETs are potentially an effective defense mechanism that neutrophils could use to mediate *M. tuberculosis* infection resistance (Figure 1B).

## Neutrophils and the role of cytokines and chemokines in inflammation in *M. tuberculosis* infection resistance

### Initial inflammation

*M. tuberculosis* infection triggers TLR signaling and induces NF-κB which mediates the induction of gene expression of pro-inflammatory cytokines such as IL-1β and TNF in neutrophils (42, 131). Inflammasomes are multimeric protein complexes and play a key role in the activation of IL-1α, and IL-1β (132). Neutrophils express components of the NOD-like receptor protein 3 (NLRP3) and absent in melanoma 2 (AIM2) inflammasomes (133). The latter are found in the cytoplasm as well as secretory and tertiary granule compartments (133). Neutrophils release IL-1β mostly in an inflammasome-dependent manner and do not release IL-1α (133). The inflammasome subunit caspase-1 activates pro-IL-1β to form IL-1β (132, 133). IL-1β activation can also occur in a caspase-1 independent manner via neutrophil proteases; NE, and proteinase 3 (PR3) (133). Furthermore, it is of interest that inflammasome components are found in neutrophil secretory vesicles. The components may play a role in phagosomal functionality or may be released into the extracellular environment and utilized by other phagocytes, but this remains to be proven in neutrophils (133).

One of the key roles of IL-1β is to mediate the release of prostaglandin E2 (PGE2), an eicosanoid. Eicosanoids are important lipid mediators derived from arachidonic acid (AA) and are rapidly synthesized by phagocytes after acute challenge with *M. tuberculosis* (134, 135). Cyclo-oxygenase-2 (COX-2) competes with 5-Lipoxygenase (5-LO) or 15-lipoxygenase (15-LO) for the generation of each of the different eicosanoids. During inflammation macrophages and other cells, including neutrophils, can produce COX-2, which converts AA to PGE2. 5-Lipoxygenase (5-LO) converts AA to LTB4 from leukotriene A4 (LTA4). PGE2 and LTB4 mainly have proinflammatory effects and mediate the rapid recruitment of neutrophils to the site of infection and inflammation (136, 137). LTB4 promotes phagocytosis and the bactericidal activity of neutrophils (136, 138, 139) (Figure 1C).

Furthermore, neutrophils are a possible source of IL-12 mediated IFN-γ release (140). However, whether this occurs through direct *M. tuberculosis* stimulation is unknown. Neutrophils release IFN-γ after stimulation by degranulating agents which is due to an available small storage of IFN-γ (140). In addition, neutrophil stimulation by IL-12 alone or in combinations with lipopolysaccharide (LPS), IL-2, IL-18, or IL-15, induces IFN-γ synthesis by neutrophils (140).

Neutrophils matured with IFN-γ have marked upregulation of multiple transcripts where Guanylate Binding Protein (GBP) showed the highest changes. GBPs are a subfamily of the IFN inducible GTPase superfamily (141, 142). GBP-5, in particular, is strongly upregulated in transcriptomes from an immature myeloid cell line (PLB-985) matured in the presence of IFN-γ (143). PLB-985 cells can differentiate into terminally mature neutrophils and have the ability to mimic the physiological conditions of stimulation (144). The exact role of GBP-5 has not been described in neutrophils yet, but it is possible that it enhances the NLRP3 inflammasome and IL-1β production, as in macrophages (143) (Figure 1C).

IFN-γ may increase the half-life of neutrophils in culture by being anti-apoptotic (143) and in this manner contributes to the pro-inflammatory state. Pathology in pulmonary tuberculosis is associated with neutrophils expressing IFN-γ and type I IFNs (145). This transcriptional signature is found in patients with active TB but infrequently in healthy individuals or those with latent TB (145). Type 1 IFNs may contribute to disease progression but the pro-inflammatory effect of IFN-γ from a neutrophil perspective may be effective for short bursts and in a setting where *M. tuberculosis* is effectively killed. The promotion of this initial pro-inflammatory state and release of TNF and IFN-γ by neutrophils is essential to effectively clear *M. tuberculosis* infection (48, 143).

### The resolution of inflammation in *M. tuberculosis* infection

Apoptosis represents a pivotal point in the control of inflammation as well as in the control of the cellular immune response (146). A delicate balance exists between apoptotic cell death, clearance of apoptotic cells and ongoing inflammatory responses (80, 147, 148). Not only does the efferocytosis of apoptotic neutrophils by tissue resident macrophages prevent spillage of neutrophil content into surrounding tissue (80, 147, 149, 150), but it also decreases pro-inflammatory mediators (148). Clearance of infection without a significant acquired immune response is favored by early killing of *M. tuberculosis* by neutrophils, followed by apoptotic neutrophil death, and an anti-inflammatory response in the phagocytosing macrophage (35, 105).

A hallmark of the anti-inflammatory response is the production of TGF-β and PGE2, and the inhibition of IL-6, IL-8, IL-12, and TNF release by the phagocytosing macrophages (151). Studies have shown that cAMP-elevating agents such as PGE2 result in increased levels of AnnexinA1 (ANXA1) (152). ANXA1, a protein found in neutrophils, stimulates release of the anti-inflammatory cytokine, IL-10, by macrophages, and inhibits neutrophil migration (153). In addition, ANXA1 promotes efferocytosis of apoptotic cells (154, 155) (Figure 1D).

In addition to the release of endogenous anti-inflammatory mediators, pro-resolution action is also required. Lipoxins, protectins, resolvins and macrophage mediator in resolving inflammation (maresins) are unique mediators fulfilling this duality (137, 156, 157). Rising PGE2 levels eventually act as a “lipid mediated class switch” by transcriptionally inducing 15-LO in neutrophils and shifting the production of PGE_2_ and LTB4 in favor of lipoxin A4 (LXA4) (158). LXA4 decreases neutrophil-mediated tissue damage, neutrophil proliferation, and adhesion, and increases efferocytosis of apoptotic neutrophils and IL-10 production by macrophages (159). Resolvins, protectins and maresins are oxygenated metabolites derived from eicosapentaenoic acid (EPA) and docosahexaenoic acid (DHA) that is biosynthesized from omega-3 essential polyunsaturated fatty acids (137, 160). Collectively resolvins, protectins, and maresins regulate neutrophil apoptosis, efferocytosis by macrophages, inhibition of pro-inflammatory cytokines, release of IL-10 by local macrophages and tissue regeneration (159) (Figure 1D).

Finally, neutrophils express inducible nitric oxide synthase (iNOS) which converts the amino acid L-arginine to L-citrulline and nitric oxide (NO). iNOS/NO limits the production of IL-1β and therefore limits further recruitment of neutrophils (34, 161, 162). It is not known to what extent these neutrophil anti-inflammatory mechanisms are at play during early encounters of PMNs with *M. tuberculosis* in the lung (Figure 1D).

## Conclusion

At first glance, the association of uncontrolled neutrophil recruitment and pathology in TB would argue against a role of these cells in *M. tuberculosis* infection resistance. However, neutrophils are multi-functional cells with variable roles in host defense. For example, there is documented inter-individual variability in the ability of neutrophils to kill *M. tuberculosis* suggesting that the role of neutrophils in an early encounter with *M. tuberculosis* may differ from the more integrated role in the presence of a strongly developed acquired immune response to the bacillus. As reviewed, the neutrophil has a large armamentarium of highly effective anti-microbial effector mechanisms that may come into play during the early stage of *M. tuberculosis* infection. Investigating the possible role of neutrophils in persons who remain free of *M. tuberculosis* infection despite documented high exposure to the bacillus offer an interesting opportunity. It may be that resisters possess a different ratio of neutrophil subpopulations, predominated by effective killers with a propensity to undergo apoptosis, compared to those who develop TB, predominated by inflammatory necrotising damage causing neutrophils. By comparing neutrophils and their anti-microbial responses from “innate resisters” with those from *M. tuberculosis* infection susceptible persons might illuminate if and how neutrophils play a protective role in the very stage of *M. tuberculosis* infection. Experiments along these lines will not only provide a better understanding of TB pathogenesis but also contribute to a better understanding of neutrophil biology in general.

## Author contributions

All authors listed have made a substantial, direct and intellectual contribution to the work, and approved it for publication.

### Conflict of interest statement

The authors declare that the research was conducted in the absence of any commercial or financial relationships that could be construed as a potential conflict of interest.
